# Risk prediction of second primary malignancies after gynecological malignant neoplasms resection with and without radiation therapy: a population-based surveillance, epidemiology, and end results (SEER) analysis

**DOI:** 10.1007/s00432-023-05046-w

**Published:** 2023-07-15

**Authors:** Jing Wang, Chan Zhang, Yaoxian Xiang, Baojuan Han, Yurong Cheng, Yingying Tong, Dong Yan

**Affiliations:** Department of Oncology, Beijing Luhe Hospital Affiliated to Capital Medical University, Beijing, 101149 China

**Keywords:** Secondary primary malignancies (SPMs), Radiation therapy (RT), Nomogram, Risk factors, SEER

## Abstract

**Purpose:**

The association between post-resection radiotherapy for primary gynecological malignant neoplasms (GMNs) and the development of secondary primary malignancies (SPMs) remains a subject of debate. This study represents the first population-based analysis employing a multivariate competitive risk model to assess risk factors for this relationship and to develop a comprehensive competing-risk nomogram for quantitatively predicting SPM probabilities.

**Materials and methods:**

In our study, data on patients with primary GMNs were retrospectively collected from the Epidemiology, Surveillance and End Results (SEER) database from 1973 to 2015. The incidence of secondary malignant tumors diagnosed at least six months after GMN diagnosis was compared to determine potential risk factors for SPMs in GMN patients using the Fine and Gray proportional sub-distribution hazard model. A competing-risk nomogram was constructed to quantify SPM probabilities.

**Results:**

A total of 109,537 patients with GMNs were included in the study, with 76,675 and 32,862 GMN patients in the training and verification sets, respectively. The competing-risk model analysis identified age, primary tumor location, tumor grade, disease stage, chemotherapy, and radiation as risk factors for SPMs in GMN patients. Calibration curves and ROC curves in both training and verification cohorts demonstrated the predictive accuracy of the established nomogram, which exhibited a good ability to predict SPM occurrence.

**Conclusions:**

This study presents the nomogram developed for quantitatively predicting SPM probabilities in GMN patients for the first time. The constructed nomogram can assist clinicians in designing personalized treatment strategies and facilitate clinical decision-making processes.

**Supplementary Information:**

The online version contains supplementary material available at 10.1007/s00432-023-05046-w.

## Introduction

Over the past few decades, substantial improvements in cancer survival rates have led to a growing population of cancer survivors (Felicetti et al. 2018). These individuals face a heightened risk of developing new malignant tumors (Araujo-Filho et al. 2021; Bostrom and Soloway 2007), attributable to a combination of lifestyle factors, genetic predisposition, and previous cancer treatments (Weir et al. 2013). Radiation therapy (RT) is a fundamental treatment modality for pelvic tumors, including malignancies of the cervix, rectum, and ovary. While RT effectively reduces tumor recurrence and significantly enhances prognosis (De Sanctis et al. 2013), it also presents long-term risks for patients, such as the progression of secondary primary malignancies (SPMs)–a rare but consequential late complication of cancer treatment (Berrington de Gonzalez et al. 2011; Cuccia et al. 2020). Recent studies, however, have demonstrated that RT may not invariably increase the risk of SPMs and could even exert a protective effect in certain cases (Wiltink et al. 2015).

In the context of Surveillance, Epidemiology, and End Results (SEER) analyses, the risk of RT-associated SPMs remains relatively high (Conway et al. 2017; Guan et al. 2021; Li et al. 2022; Moschini et al. 2019; Yi et al. 2023; Yu et al. 2022). Several recent studies have investigated the effect of RT on SPM risk in relation to primary malignant neoplasms. For instance, Wen et al. performed a retrospective review of prior studies to assess the influence of RT on the risk of secondary bladder cancer and the clinical endpoint in patients diagnosed with gynecological cancer (Wen et al. 2022b). In another investigation, Rombouts et al. examined the association between pelvic RT and the development of rectal cancer as an SPM. Their findings indicated an elevated risk of rectal cancer in patients who had previously received RT for pelvic cancer, a risk particularly pronounced in individuals treated for prostate and endometrial cancers (Rombouts et al. 2020).

The association between RT for gynecological malignant neoplasms (GMNs) and an increased risk of secondary malignant tumors at a population-based level remains a contentious issue. Consequently, this study aims to explore the impact of GMN radiotherapy on the risk of secondary malignant tumors. By utilizing the SEER database, we conducted a comprehensive analysis of SPM characteristics in GMN patients and further examined the risk factors for SPM occurrence at a population-based level. We also established a competing-risk nomogram visualization tool to aid clinicians in identifying GMN patients at a high risk of developing SPMs, thereby facilitating closer monitoring and timely treatment.

## Materials and methods

### Data sources

SEER database is the largest and most authoritative cancer database in North America (Yu et al. 2009), encompassing cancer data for nearly 30% of the population across diverse geographic regions of the United States. We extracted data for patients with GMN who underwent surgery between 1973 and 2015 using the SEER database and SEER*Stat software (version 8.4.0, http://seer.cancer.gov/). Patients diagnosed with gynecological cancer included cervical cancer (site codes C53.0–C53.9), uterine and uterus cancer (site codes C54.0–54.3, C54.8, C54.9, and C55.9), ovarian cancer (site code C56.9), and other female genital cancers (site codes C51.0-C51.9, C52.9, C57.0–C57.9, C58.9). The follow-up period for SPMs commenced six months after the diagnosis of GMNs and concluded upon the diagnosis of any SPMs, death from any cause, or after 30 years of follow-up, whichever occurred first. We set the follow-up deadline as January 1, 2016. As the extracted data are publicly accessible and de-identified, approval from the Institutional Review Committee is not required in accordance with the Human Research Protection Office.

### Data collection

In this study, nine variables were analyzed, including marital status, age, race, primary site, stage, differentiation grade, chemotherapy, tumor size (mm), and RT. Patients who met any of the following criteria were excluded: (1) non-histological diagnoses; (2) autopsy or death certificate diagnosis; (3) no history of surgical resection; (4) age less than 18 years old; (5) no racial information provided for the patient; and (6) no complete prognostic information available. To establish and validate the nomogram, we divided enrolled patients into a training cohort and a validation cohort randomly. The detailed flow chart is illustrated in Fig S1. The final study sample comprised 109,537 patients. Using a 7:3 ratio, we assigned GMN patients to either a development group (n = 76,675) or a validation group (n = 32,862) randomly. The development group was employed to determine risk prediction factors and construct models, while the validation group was used for internal model validation.

### Statistical analysis

Categorical variables were compared as percentages using the Fisher's exact test and Chi-square test. In this study, the Mann–Whitney test was employed to analyze continuous variables with both normal and non-normal distributions. SEER*Stat 8.4.1 was used to estimate standardized incidence rates (SIRs), and SIR represented the ratio of observed SPMs to expected cases in the general population of the United States. Results were stratified by radiotherapy, age, and calendar time. The cumulative incidence of SPM development was evaluated using Fine-Gray competitive risk regression analysis. We utilized the Kaplan–Meier curve to depict the survival characteristics of patients with GMNs. A multivariate Fine and Gray proportional competing risk model was employed to identify relevant risk factors for SPM occurrence, subsequently constructing a risk prediction nomogram. The nomogram was verified by evaluating its discrimination and calibration capabilities using internal (training) and external (validation) sets, respectively. To assess prognostic accuracy, receiver operating characteristic (ROC) curves and calibration curves based on time and area under curves (AUCs) were generated at 5, 10, and 20 years.

## Results

### Demographics and clinical characteristics

We divided GMN patients into two cohorts based on the initial treatment method. The RT group comprised GMN patients who underwent neoadjuvant external beam radiotherapy and surgery, while the non-RT cohort included patients who only underwent surgery. Patients who underwent other kinds of RT (e.g., combination therapy, brachytherapy) were excluded. Between 1973 and 2015, 109,537 eligible subjects were diagnosed with GMNs, of which 14,794 patients (13.5%) were treated with RT for primary GMN cancer. Among the subjects, 88,605 patients (80.9%) were White, and the median age was 59 years (interquartile range, 49–68 years). The median follow-up time was 100 months (interquartile range, 41–200 months). The non-radiation group consisted of 94,743 patients (86.5%), while the radiation group had 14,794 patients (13.5%). Table 1 displays the baseline characteristics of cancer patients by treatment method. After a six-month incubation period, 8,407 patients (8.9%) in the non-radiation group and 1,645 patients (11.1%) in the radiation group developed SPMs.

**Table 1:** Patient demographics and pathological characteristics

	NRT	RT	*P* value
	(*N* = 94,743)	(*N* = 14,794)	
Age at GMN diagnosis, y			
< 50	24,995 (26.4%)	2873 (19.4%)	< 0.001^b^
50–69	49,222 (52.0%)	8036 (54.3%)	
≥ 70	20,526 (21.7%)	3885 (26.3%)	
Age at GMN diagnosis, y			
Mean (SD)	58.0 (14.3)	60.6 (13.1)	< 0.001^a^
Median [IQR]	59.0 [18.0, 99.0]	61.0 [18.0, 98.0]	
Marital. status			
Unmarried	39,924 (42.1%)	6704 (45.3%)	< 0.001^b^
Married	51,040 (53.9%)	7670 (51.8%)	
Unknown	3779 (4.0%)	420 (2.8%)	
Race			
White	76,681 (80.9%)	11,924 (80.6%)	< 0.001^b^
Black	8822 (9.3%)	1634 (11.0%)	
Other	9240 (9.8%)	1236 (8.4%)	
Grade			
Grade I/II	46,734 (49.3%)	6737 (45.5%)	< 0.001^b^
Grade III/IV	22,375 (23.6%)	5105 (34.5%)	
Unknown	25,634 (27.1%)	2952 (20.0%)	
Tumor-size			
< 2 cm	2979 (3.1%)	100 (0.7%)	< 0.001^b^
≥ 2 cm	80,893 (85.4%)	11,595 (78.4%)	
Unknown	10,871 (11.5%)	3099 (20.9%)	
Primary. Site			
Cervix uterus	10,644 (11.2%)	2299 (15.5%)	< 0.001^b^
Corpus and uterus	49,137 (51.9%)	10,364 (70.1%)	
Ovary	28,876 (30.5%)	1176 (7.9%)	
Other	6086 (6.4%)	955 (6.5%)	
Chemotherapy			
No/unknown	69,168 (73.0%)	10,848 (73.3%)	0.419^b^
Yes	25,575 (27.0%)	3946 (26.7%)	
Stage			
Localized	63,161 (66.7%)	7313 (49.4%)	< 0.001^b^
Regional	8842 (9.3%)	5059 (34.2%)	
Distant	21,324 (22.5%)	2097 (14.2%)	
Unknown	1416 (1.5%)	325 (2.2%)	
Survival. months			
Mean (SD)	134 (113)	130 (114)	< 0.001^a^
Median [IQR]	100 [6.00, 515]	97.0 [6.00, 515]	
Patients who developed SPMs	8407 (8.9%)	1645 (11.1%)	< 0.001^b^

*IQR* interquartile range, *GMN* gynecological malignant neoplasm. *SPMs*, secondary primary malignancies

^a^*P* values were calculated using the Mann–Whitney test for continuous variables and χ^2^ test

^b^*P* values were calculated using the Mann–Whitney test for categorical variables

**Fig. 1:**
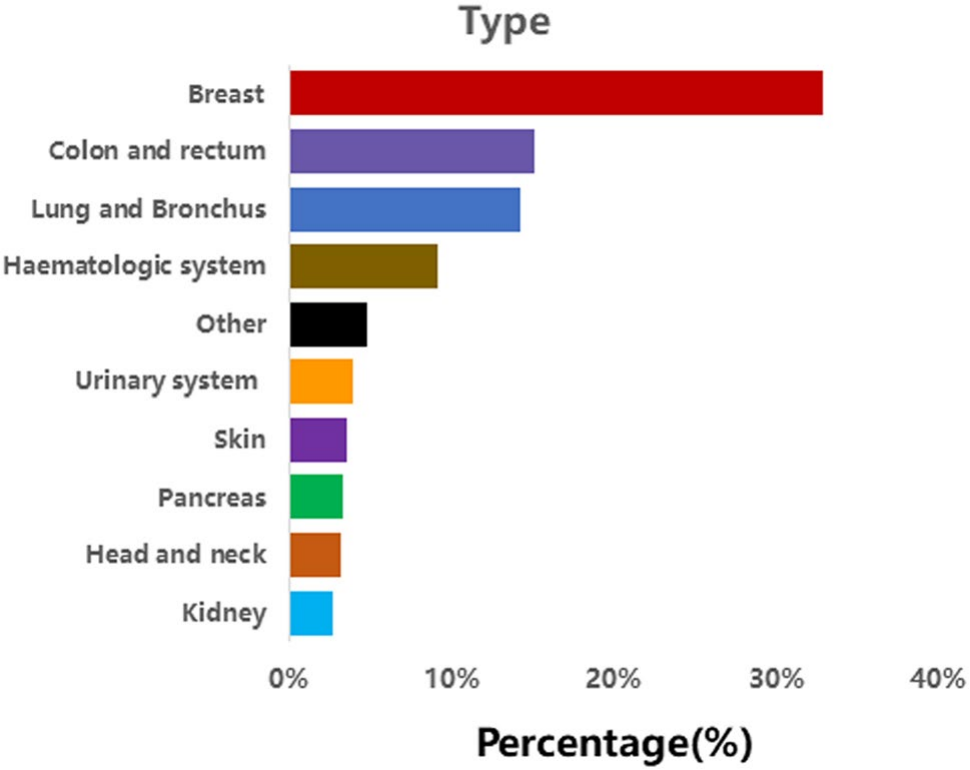
The ten most common areas of secondary primary malignancies (SPMs) in female patients

Figure 1 presents the 10 most common cancer sites of SPMs among GMN patients. The most common areas include breasts, colon and rectum, and lungs (bronchi) (Fig. 1).

### Standardized incidence ratios

Compared to the general population in the United States, patients with a record of primary GMNs have a higher risk of SPMs. In the group of patients who did not receive RT treatment (SIR: 0.94, CI: 0.92–0.95), the SIR for patients treated with RT was 1.16 (CI: 1.13–1.20), indicating an increased risk due to RT treatment. The risk for patients who did not receive RT treatment was similar to that of the general population in the United States. Among patients with a history of RT, 22 out of 100,000 patients experience excessive risk per year.

### Survival and cumulative incidence of SPMs

**Fig. 2:**
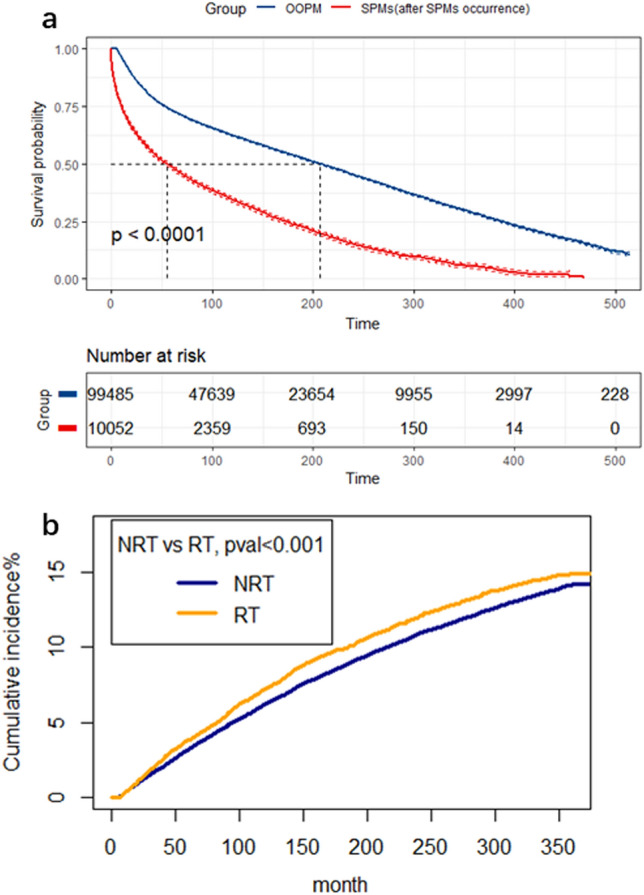
OS curves for gynecological malignant neoplasms (GMNs) patients from the only one primary malignancy (OOPM) cohort and the SPMs’ cohort (after SPMs’ occurrence) (**a**), Comparison of cumulative incidence of second primary malignancies (SPMs) between patients receiving Radiation Therapy (RT) and those not Radiation Therapy (NRT) (**b**)

The 5-,10-, and 20-year overall survival (OS) rates for GMN patients in the SPMs cohort vs. the only one primary malignancy (OOPM) cohort were 87.7% vs. 73.1%, and 71.9% vs. 62.3%, 41.6% vs. 45.2%, respectively. No significant difference was found in OS between the two cohorts (S2 Fig). Furthermore, after the occurrence of SPMs, GMN patients in the SPMs group had evidently worse OS than those in the OOPM group. (Fig. 2a): the 5-, 10-, and 20-year OS since their SPMs' diagnosis were only 48.4%, 34.4% and15%. Next, we used the competitive risk F-G test to analyze the overall cumulative incidence of SPMs diagnosed as GMNs, in which death was regarded as a competitive event. The 5-, 10-, and 20-year cumulative incidence of SPMs in GMN patients receiving RT treatment were 3.84%, 7.27% and 12.06%, respectively; the cumulative incidence without radiotherapy was 3.23%, 6.27% and 10.93% (P < . 001) (Fig. 2b).

### Predictors of SPMs occurrence

A multivariable Fine and Gray's proportional risk model was employed to assess relevant risk factors which were correlated with the progression of second primary malignancies (SPMs) (Table 2). Independent risk factors for SPM occurrence included older age (age 50–69 years vs. age < 50 years, hazard ratio (HR) = 1.747, 95% CI 1.649–1.851; age ≥ 70 years vs. age < 50 years, HR = 1.458, 95% CI 1.361–1.563), primary site (corpus and uteri vs. cervix, HR = 1.209, 95% CI 1.120–1.304; ovary vs. cervix, HR = 1.270, 95% CI 1.165–1.384; other sites vs. cervix, HR = 1.233, 95% CI 1.111–1.369), and the performance of radiotherapy (vs. not performed, HR = 1.114, 95% CI 1.053–1.177). Independent protective factors included the performance of chemotherapy (vs. not performed, HR = 0.784, 95% CI 0.730–0.842), stage (regional vs. localized, HR = 0.861, 95% CI 0.804–0.921; distant vs. localized, HR = 0.496, 95% CI 0.456–0.541; unknown vs. localized, HR = 0.929, 95% CI 0.806–1.070), and grade III/IV (vs. grade I/II, HR = 0.864, 95% CI 0.815–0.917). In subgroup analysis, the risk of SPMs is associated with RT, with HR > 1 in most subgroups (S3 Fig.).

**Table 2:** Multivariable competing risk regression analysis of risk of developing SPMs in GMNs

Characteristics	Multivariable competing risk regression	
	HR (95% Cl)	*P*
Age at GMN diagnosis		
< 50	1	
50–69	1.747 (1.649 1.851)	< 0.001
≥ 70	1.458 (1.361 1.563)	< 0.001
Marital. status		
Unmarried	1	
Married	1.018(0.977 1.061)	0.48
Unknown	1.018(0.912 1.136)	0.79
Race		
White	1	
Other	0.010(0.940 1.085)	0.82
Black	1.009(0.940 1.083)	0.83
Stage		
Localized	1	
Regional	0.861(0.804 0.921)	< 0.001
Distant	0.496(0.456 0.541)	< 0.001
Unknown	0.929(0.806 1.070)	0.39
Primary. Site		
Cervix uterus	1	
Corpus and uterus	1.209(1.120 1.304)	< 0.001
Ovary	1.270(1.165 1.384)	< 0.001
Other	1.233(1.111 1.369)	< 0.001
Tumor-size		
< 2 cm	1	
≥ 2 cm	0.906(0.816 1.006)	0.12
Unknown	0.980(0.877 1.095)	0.76
Grade		
Grade I/II	1	
Grade III/IV	0.864(0.815 0.917)	< 0.001
Unknown	0.959(0.911 1.008)	0.17
Chemotherapy		
No/unknown	1	
Yes	0.784(0.730 0.842)	< 0.001
Radiation therapy		
No	1	
Yes	1.114(1.053 1.177)	< 0.001

*GMN* gynecological malignant neoplasm, *SPMs* secondary primary malignancies

### Nomogram construction

**Fig. 3:**
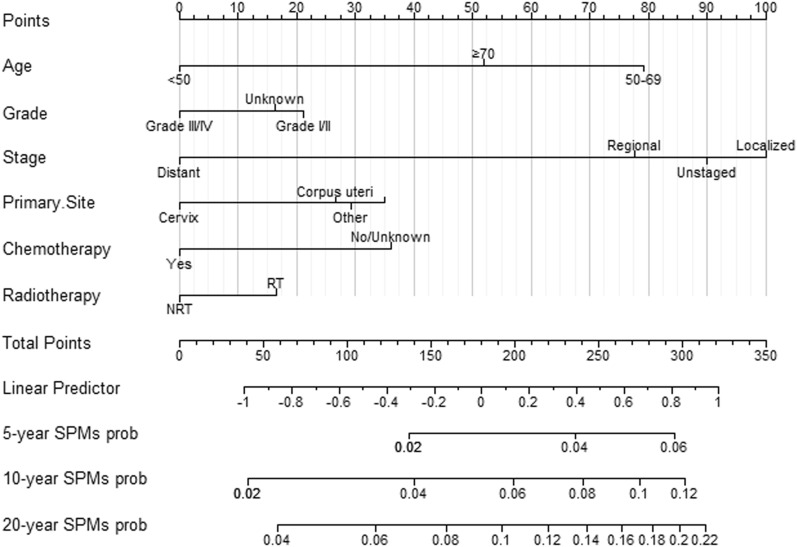
Competitive risk column chart for predicting the 5-year, 10-year, and 20-year SPMs probabilities of GMNs patients

Multivariate Fine and Gray's risk analysis demonstrated that grade, age, primary site, stage, chemotherapy and radiotherapy were independent risk predictors of SPMs. Based on these independent factors, nomograms illustrating the 5-, 10-, and 20-year probabilities of SPMs were constructed (Fig. 3). By summing the scores for each selected variable, the probability of SPMs for an individual cancer patient can be well evaluated.

### Nomogram validation

**Fig. 4:**
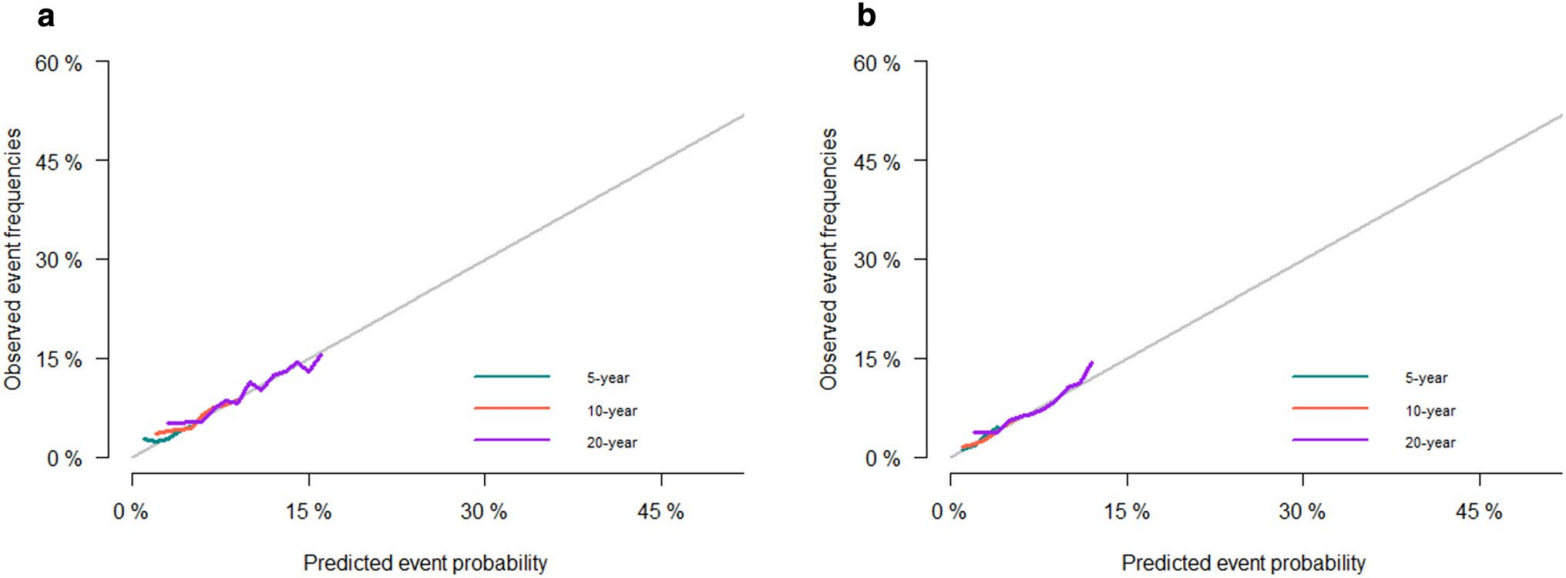
Calibration curves of the competing-risk nomogram for predicting 5-, 10-, and 20-year SPMs’ probabilities in the training cohort (**a**) and validation cohort (**b**)

The calibration diagrams confirm the best agreement between nomogram-predicted development set (Fig. 4a) and the validation set **(**Fig. 4b) and observed probabilities. According to the development verification queue, the calibration curve based on the prediction factor of the column chart indicates that the occurrence of SPMs is very consistent with the observed results. In terms of the 5-, 10-, and 20-year ROC curve and the AUC of SPMs' probabilities, the training cohort’s AUC values were 0.575, 0.601, and 0.621 (Fig S3a), and the validation cohort’s AUC values were 0.59, 0.609, and 0.625 (Fig S3b). Considering the calibration diagrams and the AUC value, it can be concluded that the nomogram has sufficient discriminative ability and can make accurate predictions.

### Clinical application of nomograms

**Fig. 5:**
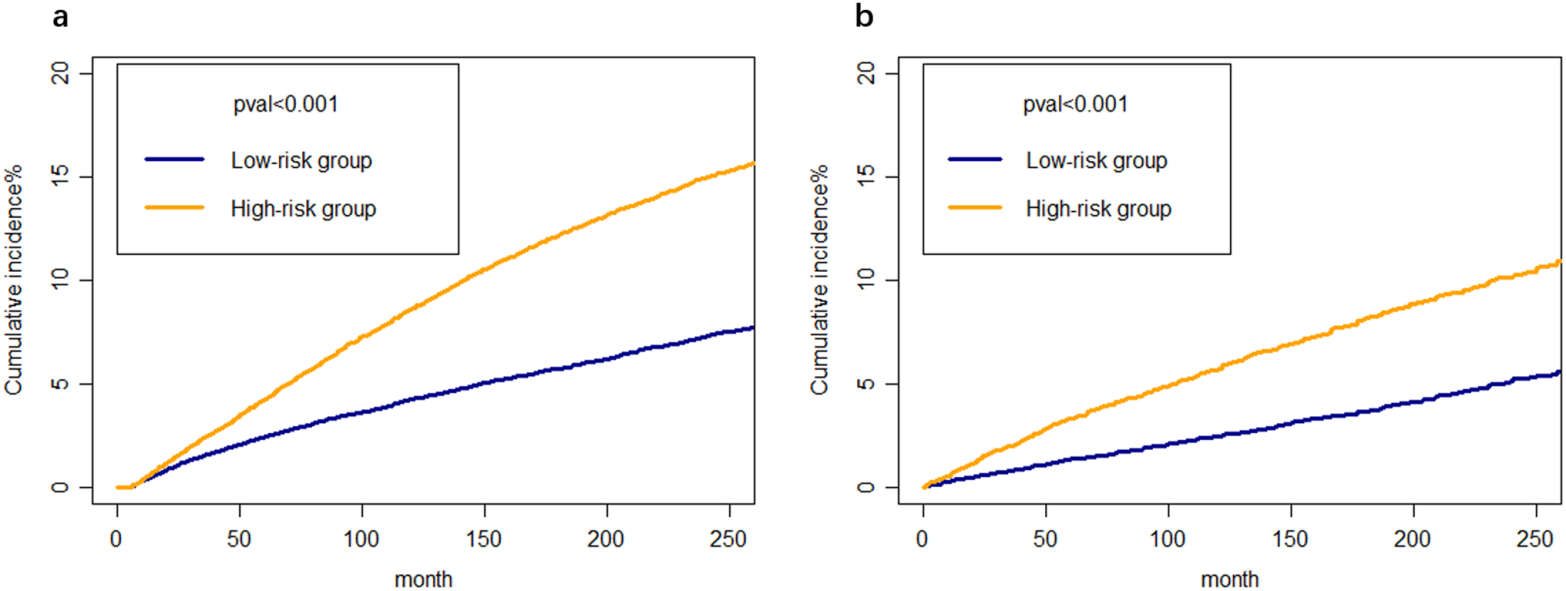
Cumulative incidence curves of SPMs in the low-risk and high-risk groups of training cohort (**a**) and validation cohort (**b**)

Effective patient management necessitates appropriate risk stratification. By summing the scores for each patient, a risk score is generated. Patients with GMNs were classified into two groups based on the median individual risk score (208 points) derived from the competing-risk nomogram in the training cohort: the high-risk group (≥ 208 points) and the low-risk group (0–208 points). As depicted in Fig. 5a, b, the cumulative incidence of SPMs in the high-risk group was evidently greater than that in the low-risk group for both the training and verification groups.

## Discussion

This study aims to explore the correlation between the development of SPMs following GMN resection and radiotherapy, as well as the subsequent impact on the prognosis of SPMs. The current data reveals that the cumulative incidence of SPMs in GMN patients who previously received radiotherapy is evidently higher than in those who did not undergo radiotherapy. Radiotherapy has been established as an independent risk characteristic for SPMs among GMN survivors. Additionally, this population-based cohort study identified the characteristics and risk factors of SPMs in GMNs.

An increase in cancer survivorship, the long-term adverse reactions of radiotherapy, advancements in early screening and diagnostic technologies, and the ongoing influence of risk factors have all contributed to a substantial rise in the incidence of various malignant tumors (Viyanant and Upatham 1988). An increasing amount of research is concentrating on the location of SPMs after the first primary malignant tumor. Ding et al. conducted a retrospective study on 11,017 patients with colorectal neuroendocrine neoplasms, discovering that the most common sites of SPM in males were the prostate, lungs (bronchi), rectum, and kidneys. In females, the most prevalent locations were the breasts, lungs (bronchi), rectum, and uterine body (Ding et al. 2023). Conversely, Warschkow et al. examined a cohort of 77,484 patients who had undergone resection of localized or locally advanced rectal adenocarcinoma, finding that prostate, breast, and lung cancers were the three most common types of SPMs (Warschkow et al. 2017). By analyzing extensive data from the SEER database, focusing on primary GMNs, it was determined that the three most frequent SPM sites in female GMN patients were the breast, colorectal, and lung (bronchus) regions. This underscores the importance of emphasizing cancer screening in these areas during GMN patient follow-up.

In summary, our study highlights that research on the risk factors for SPMs in GMNs is limited, but our analysis identified age, primary site, grade, stage, chemotherapy, and radiotherapy as significant risk factors for developing SPMs in GMNs. Among these factors, radiotherapy was found to be a key contributor to the occurrence of SPMs in GMNs. Our finding is consistent with past studies which reported a higher risk of developing SPMs in patients undergoing radiotherapy for other tumor types such as gynecological, rectum, and prostate cancers (Guan et al. 2021; Li et al. 2022; Moschini et al. 2019; Wen et al. 2022a). Generally, secondary cancers tend to occur in irradiated or adjacent areas, with the probability of random effects such as carcinogenesis increasing with the increasing dose of radiotherapy (Brown et al. 2010; Rombouts et al. 2018). The introduction of intensity-modulated RT (IMRT) has shown benefits in various tumor sites (Klem et al. 2008), as it allows for different doses to be delivered to different structures under the same irradiation (Wang et al. 2006). However, there is limited data supporting the relationship between the distribution of radiotherapy doses and the increased risk of secondary malignant tumors (Berrington de Gonzalez et al. 2015; Lonn et al. 2010). Our study also found that chemotherapy acted as a protective factor for GMNs to develop SPMs, consistent with previous research (Guan et al. 2021). Poor histopathology grading and distant metastasis were negatively related to the occurrence of SPMs in GMNs, which aligns with the results of Bateni SB et al. for patients with neuroendocrine tumors (Bateni et al. 2021). Our study also revealed that the presence of primary sites in the cervix reduced the risk of developing SPMs in patients with ovarian cancer. This difference may be attributed to the distinct clinical pathological characteristics of different primary organs (Lazzaroni et al. 2022), further emphasizing the diversity of different primary lesions.

This study presents several advantages and limitations. The advantages include the use of the SEER database, which provides detailed clinicopathological information about GMNs, and the construction of a competitive risk nomogram based on the multivariate Fine and Gray proportional sub-distribution risk model. This nomogram serves as a practical tool for improving guidance, monitoring, and managing GMN survivors and is the first model to assess the probability of SPMs occurring in survivors of GMN at 5, 10, and 20 years after the first diagnosis. The effectiveness of the nomogram was validated using ROC curves and calibration curves, allowing for proactive screening methods and close follow-up for potential SPMs in individuals with GMNs with a total risk point of 208 or above.

However, there are some limitations to consider. Firstly, there is a selection bias in the retrospective study, and future prospective studies will be necessary to verify the nomogram. Secondly, the occurrence of SPMs may be influenced not only by radiotherapy but also by other key risk factors, including lifestyle, environmental factors, genetic background, and other treatments that are not considered in our work owing to the unavailability of relevant data in the SEER dataset. Lastly, the SEER dataset only records the initial treatment strategy of GMN, and it is unclear whether GMN patients will receive delayed radiation exposure in further treatment, which could lead to misclassification of patients in the RT group as non-RT group. Despite these limitations, the main conclusion of our study remains valid, although the increased risk of RT may be underestimated.

## Conclusion

Utilizing a large population from SEER database, our work identified primary site, age, grade, stage, chemotherapy, and radiotherapy as independent risk factors for SPMs in GMN patients. These characteristics were found to be correlated with the progression of SPMs in GMN patients. We constructed a competitive risk nomogram based on a competing-risk model to predict the 5-, 10-, and 20-year probabilities of SPMs in GMN patients, demonstrating strong predictive capabilities.

## Supplementary Information

Below is the link to the electronic supplementary material.

Supplementary file1 (DOCX 104 kb)

Supplementary file2 (DOCX 128 kb)

Supplementary file3 (DOCX 182 kb)

Supplementary file4 (DOCX 247 kb)

## Data Availability

The data sets used and/or analyzed during the current study are available from the Surveillance, Epidemiology, and End Results (SEER) databasehttp://seer.cancer.gov/.
