# Supplementary material for: Risk prediction of second primary malignancies after gynecological malignant neoplasms resection with and without radiation therapy: a population-based surveillance, epidemiology, and end results (SEER) analysis
Source: J Cancer Res Clin Oncol. 2023 Jul 15;149(14):12703–11. doi: 10.1007/s00432-023-05046-w (PMC10587290; doi:10.1007/s00432-023-05046-w)
Supplement: Supplementary file 1 — Supplementary file1 (DOCX 104 kb) [file 432_2023_5046_MOESM1_ESM.docx]

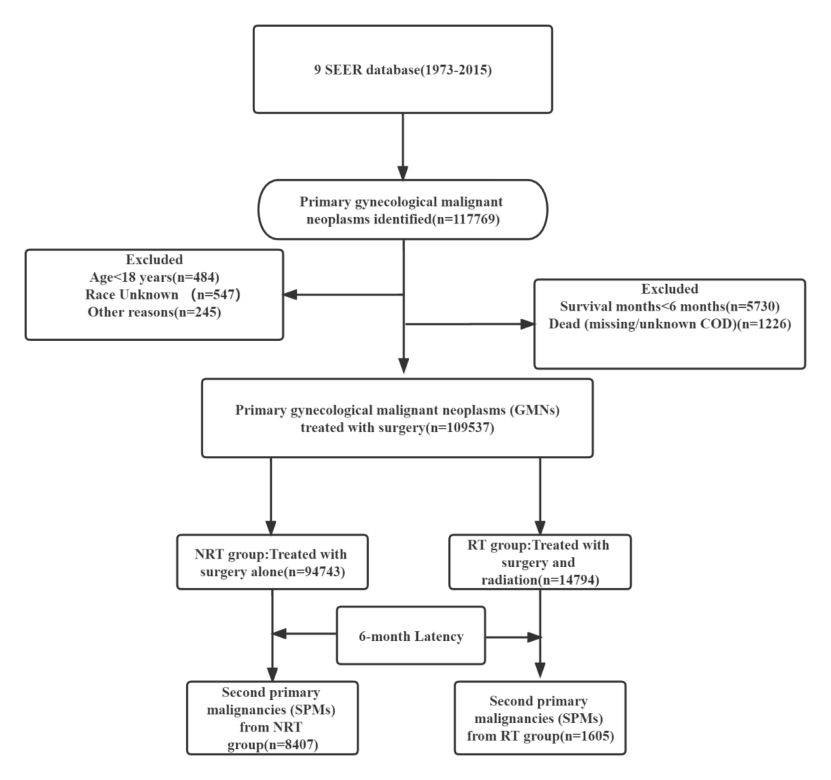


S1 Fig. Flow diagram.

Abbreviations: *RT*, radiation therapy; *NRT*, no radiation therapy; *SEER*, Surveillance, Epidemiology and End Results; *PSM*, propensity score matching
