# Supplementary material for: Risk prediction of second primary malignancies after gynecological malignant neoplasms resection with and without radiation therapy: a population-based surveillance, epidemiology, and end results (SEER) analysis
Source: J Cancer Res Clin Oncol. 2023 Jul 15;149(14):12703–11. doi: 10.1007/s00432-023-05046-w (PMC10587290; doi:10.1007/s00432-023-05046-w)
Supplement: Supplementary file 2 — Supplementary file2 (DOCX 128 kb) [file 432_2023_5046_MOESM2_ESM.docx]

S2 Fig

OS curves for primary gynecological malignant neoplasms (GMNs) from the only one primary malignancy (OOPM)cohort and the secondary primary malignancies (SPMs’ ) cohort (from initial primary cancer diagnosis)
