# Supplementary material for: Risk prediction of second primary malignancies after gynecological malignant neoplasms resection with and without radiation therapy: a population-based surveillance, epidemiology, and end results (SEER) analysis
Source: J Cancer Res Clin Oncol. 2023 Jul 15;149(14):12703–11. doi: 10.1007/s00432-023-05046-w (PMC10587290; doi:10.1007/s00432-023-05046-w)
Supplement: Supplementary file 3 — Supplementary file3 (DOCX 182 kb) [file 432_2023_5046_MOESM3_ESM.docx]

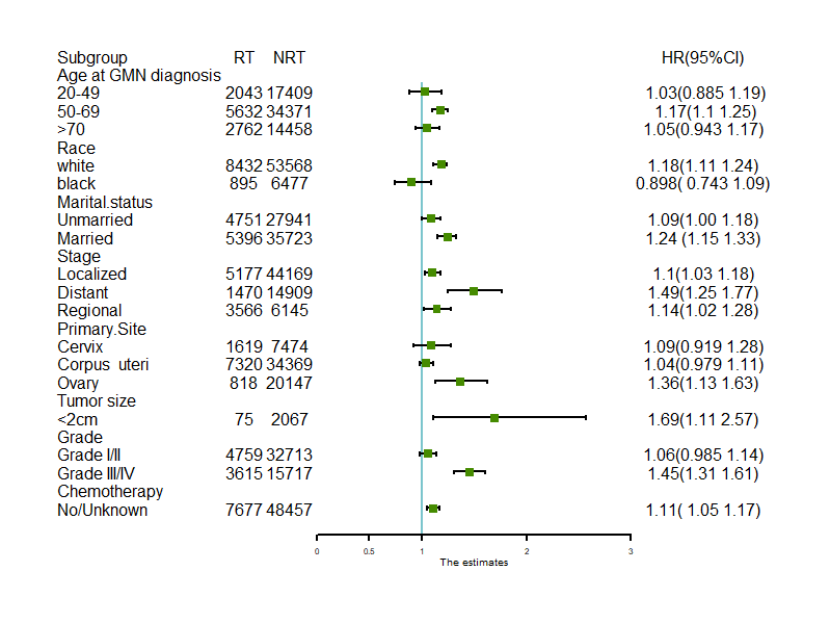


S3Fig.

Subgroup analyses of competing risk regression for the risk of developing secondary primary malignancies (SPMs)
